# Lifestyle medicine curriculum for a preventive medicine residency program: implementation and outcomes

**DOI:** 10.3402/meo.v21.29339

**Published:** 2016-08-08

**Authors:** Haq Nawaz, Paul V. Petraro, Christina Via, Saif Ullah, Lionel Lim, Dorothea Wild, Mary Kennedy, Edward M. Phillips

**Affiliations:** 1 Department of Preventive Medicine, Griffin Hospital, Derby, CT, USA; 2 Department of Internal Medicine, Yale University School of Medicine, New Haven, CT, USA; 3 Institute of Lifestyle Medicine, Physical Medicine & Rehabilitation Service, VA Boston Healthcare System, Brockton, MA, USA

**Keywords:** lifestyle medicine, preventive medicine, medical education

## Abstract

**Background:**

The vast majority of the healthcare problems burdening our society today are caused by disease-promoting lifestyles (e.g., physical inactivity and unhealthy eating). Physicians report poor training and lack of confidence in counseling patients on lifestyle changes.

**Objective:**

To evaluate a new curriculum and rotation in lifestyle medicine for preventive medicine residents.

**Methods:**

Training included didactics (six sessions/year), distance learning, educational conferences, and newly developed lifestyle medicine rotations at the Institute of Lifestyle Medicine, the Yale-Griffin Prevention Research Center, and the Integrative Medicine Center. We used a number of tools to assess residents’ progress including Objective Structured Clinical Examinations (OSCEs), self-assessments, and logs of personal health habits.

**Results:**

A total of 20 residents participated in the lifestyle medicine training between 2010 and 2013. There was a 15% increase in residents’ discussions of lifestyle issues with their patients based on their baseline and follow-up surveys. The performance of preventive medicine residents on OSCEs increased each year they were in the program (average OSCE score: PGY1 73%, PGY2 83%, PGY3 87%, and PGY4 91%, *p*=0.01). Our internal medicine and preliminary residents served as a control, since they did participate in didactics but not in lifestyle medicine rotations. Internal medicine and preliminary residents who completed the same OSCEs had a slightly lower average score (76%) compared with plural for resident, preventive medicine residents (80%). However, this difference did not reach statistical significance (*p*=0.11).

**Conclusion:**

Incorporating the lifestyle medicine curriculum is feasible for preventive medicine training allowing residents to improve their health behavior change discussions with patients as well as their own personal health habits.

Lifestyle medicine is the evidence-based practice of helping individuals and families adopt and sustain healthy behaviors that can affect health and quality of life (1). The World Health Organization estimates that by 2020, two-thirds of all disease will be the result of lifestyle choices such as overeating, inactivity, and smoking (2). Nutrition, physical activity, and obesity are among the 12 Leading Health Indicators, a set of high priority public health issues in the United states are Physical Activity, Overweight, Obesity, and Tobacco Use and Substance Abuse (3). The WHO labeled the American environment as ‘obesogenic’ with unhealthy diets, increasing food portion sizes, and markedly decreasing physical activity (2).

Patients view physicians as their most reliable source of health information (4, 5), and when counseled, they are more likely to make changes in diet and physical activity (6). Despite the United States Preventive Services Task Force recommendation (7, 8), the majority of healthcare providers do not routinely screen or assist their patients in modifying disease-promoting lifestyles (9–11). An important reason for this is inadequate medical education and the physician's lack of confidence in lifestyle medicine skills (12–14). Physicians are also reluctant to counsel on behaviors that they do not personally pursue (6, 15). Very few residency training programs currently offer formal training in lifestyle medicine. The current training structure emphasizes diagnostic and pharmacologic approaches to disease management and de-emphasizes health-promoting lifestyle interventions as a valid, effective and rationale treatment in primary and secondary prevention of chronic diseases (16).

To date, there have been no reported systematic and comprehensive efforts to incorporate a lifestyle medicine curriculum into residency training programs. The Preventive Medicine Residency Program at Griffin Hospital has developed and implemented an outcomes assessment of a lifestyle medicine curriculum in its program.

## Methods

### Setting and participants

The Griffin Hospital is a 160-bed academic community hospital located in southern Connecticut. Griffin Hospital offers two Accreditation Council for Graduate Medical Education (ACGME) accredited residency programs in internal medicine (categorical medicine (total 12 residents/year), preliminary medicine (9 positions/year)) and preventive medicine. The preventive medicine residency is offered in conjunction with internal medicine in a combined internal medicine/preventive medicine program with a total of 12 positions. The combined internal medicine/preventive medicine residency program is affiliated with the Yale School of Medicine, the Yale School of Public Health, and the Yale-Griffin Prevention Research Center.

### Needs assessment

In December 2009, we conducted an Objective Structured Clinical Examination (OSCE) (17) with three patient communication stations, including preventive counseling and patient-centered care to assess residents’ competency in lifestyle medicine skills. Scoring was done using the validated ACGME-endorsed Calgary–Cambridge instrument, and a score of 60% or above was considered a passing score (18). About 50% of the residents did not achieve a passing score. None of the residents were adequately competent in behavioral counseling or motivational interviewing. While residents were able to recognize health risk behaviors, they lacked skills in motivational interviewing or applying tools of lifestyle medicine such as prescribing exercise. These findings led the Griffin Department of Medical Education to develop and implement a lifestyle medicine curriculum. This curriculum was supported through a preventive medicine residency training grant from Health Resources, and Service Administration, grant number D5HHP18960. The project was functional from September 2010 to August 2013.

### Training

Training included didactics, distance learning, educational conferences, and a newly developed lifestyle medicine rotation at the Institute of Lifestyle Medicine (ILM) (19). We also provided additional training opportunities at the Yale-Griffin Prevention Research Center (20), and the Integrative Medicine Center (IMC) at Griffin Hospital. The curriculum spanned the entire residency and covered both clinical and practicum phases. Some components of the didactic training were repeated each year to ensure that most residents could benefit from these sessions. The curriculum included a 1-h didactic training session on lifestyle medicine every other month starting in year 1 of the project (six sessions/year, in person and/or via web-conferencing), as well as interactive sessions on personality testing and coaching. Topics included healthy diet, physical activity, smoking cessation, stress reduction, motivational interviewing, counseling strategies, lifestyle coaching skills, integrative medicine, and behavioral counseling. These sessions were led by faculty from the ILM and Yale-Griffin Prevention Research Center. The lectures were complemented by web-based modules (stress management, weight management, prescribing exercise, osteoporosis, metabolic syndrome, acute low back pain) from the Harvard Medical School Department of Continuing Education (written by ILM faculty). Sessions provided overview of epidemiology of key lifestyle issues such as obesity, sedentary lifestyle, stress, and metabolic syndrome followed by discussion of risk factors and predictors of unhealthy behavior. Sessions were highly interactive and included training in motivational interview with practical tips and role plays for promoting behavior change among patients such as using exercise prescriptions for patients.

In the lifestyle medicine rotation, residents received exposure to a broad range of lifestyle medicine skills to complement their training (see Table 1). The ILM was founded in 2007 at the Spaulding Rehabilitation Hospital and Harvard Medical School. The educational objectives included: 1) understanding lifestyle medicine and recognizing how LM can be practiced in a variety of clinical settings; 2) understanding the competencies in lifestyle medicine; 3) understanding the importance of physician health and its role in counseling patients; 4) learning how to incorporate lifestyle medicine into clinical practice; 5) understanding the components of the Medicare Wellness Visit; and 6) gaining a basic understanding of the business side of lifestyle medicine. Each resident rotation was offered as a 2-week long required rotation at the ILM in Boston. During the rotation, residents shadowed their preceptors and engaged in encounters involving lifestyle-related issues. Residents also worked with a lifestyle coach to improve their own personal health habits. During rotation, residents were required to spend time in the gym and complete self-assessment surveys on their approach to lifestyle medicine. Residents completed reading assignments from the LM literature, actively engaged in coaching patients, underwent individual exercise testing, participated and observed mindfulness-based stress management, participated in patient-centered counseling workshop, and observed physicians practicing LM. Residents also received personalized coaching sessions on a lifestyle behavior of their choice during the rotation so that they could experience the challenge in changing a lifestyle medicine behavior themselves. Residents were asked to prepare a case-based presentation at the end of the rotation and to report on their personal health behavior change experience during the rotation.

**Table 1:** Description of the core content areas included in the lifestyle medicine rotation

Lifestyle medicine rotation content area	% of rotations included
Health coaching/patient-centered care: skills necessary to develop a patient-centered clinical relationship	100
Lifestyle medicine in practice: observations of clinicians who've integrated lifestyle medicine into their existing clinical practices	100
Personal fitness assessment: hands-on session to assess fitness level and develop a personal fitness plan for the resident	100
Stress management: cognitive behavioral therapy techniques to manage a stressed patient	100
Patient fitness assessments: a hands-on guide to office-based fitness assessments for patients	90
Business of lifestyle medicine: discussion of how to make lifestyle medicine financially viable in the current medical system	80
Personal health coaching: hands-on coaching session to elicit a lifestyle modification for the resident	80
Benson–Henry Mind Body Medicine Institute: observation and discussion of mind–body medicine techniques and programs	70
Patient-centered medical home: observation of a cutting edge clinical practice	70
Nutrition assessment: a guide to basic program planning, counseling, and metabolic assessments	50

Additional areas supplementing the core	

Physical activity in older adults	40
General lifestyle medicine discussions; self-determination and willpower in behavior change; observation of shared medical appointments	20
Employee wellness in a medical setting, general behavioral health, positive psychology, medical weight management, observation of a lifestyle medicine clinic	10

Another rotation the residents could participate in was at the Yale-Griffin Prevention Research Center and included completing an elective 4-week research rotation in diet, nutrition, and obesity. Objectives included learning the role of diet, nutrition, and supplements in preventing and treating disease, becoming proficient in searching published literature, conducting systematic reviews, and participating in the design of research studies.

In addition, residents were given the opportunity to complete a 2-week elective at the IMC to understand the epidemiology of the most commonly used Complementary and Alternative Medicine (CAM) therapies as well as patterns of their use, licensing, legal status, and clinical applications, and to participate in dietary supplement research. The IMC was created to bridge the gap between conventional and alternative medicine for a broad range of medical conditions. It is founded on the principles of patient-centered care and evidence-based medicine. The patient is provided with evaluations that are holistic (consider the whole person) by doctors specializing in internal and preventive medicine, a nurse practitioner, and two naturopathic physicians with expertise in a wide array of natural, complementary, and alternative therapies. During this rotation, residents had the opportunity to participate in designing and conducting research studies involving dietary supplements and micronutrients, as well as epidemiology, patterns of use, licensing, legal status, and clinical applications of the most commonly used CAM therapies.

Residents were provided funds to attend two live ILM conferences in Boston (‘Lifestyle medicine: Tools for Promoting Healthy Change’ and ‘Active Lives: Transforming Ourselves and Our Patients’) that were conducted by the ILM (www.harvardlifestylemedicine.org/).

### Personal habits improvement

Our residents were encouraged to maintain a personal diary to reflect on their current lifestyle habits and to think of ways to improve their lifestyle. We also encouraged personal sharing during regular faculty–resident group meetings. Furthermore, we provided pedometers (Fitbit^®^) to our residents, which included a social network component that enabled residents to monitor their individual activity levels and compare it with that of others. Preventive medicine residents also organized a walk in the local community themed ‘Healthy Doctors–Healthy Patients’. In addition, we implemented brief fitness activities that included bursts of high-intensity exercises followed by stretching and relaxation after regularly scheduled preventive medicine noon conferences, led by a preventive medicine resident.

### Evaluations

In order to evaluate the residents training in lifestyle medicine, a number of tools were used to assess their progress including self-assessment and OSCEs.

### Objective Structured Clinical Examination

Videotaped OSCE (17) assessments with standardized patients (trained paid actors) were conducted in the fall and spring of each year to assess residents’ competency in lifestyle medicine and behavioral counseling skills. As part of the OSCE, the Calgary–Cambridge patient-centered assessment tool (18), which assesses 28 explicit behaviors and skills (such as greeting, introductions, respect, negotiating agenda, etc.), was used to evaluate residents’ communication skills. The score was reported as a percentage of the maximum possible score of forty-two. Lifestyle medicine scenarios for the OSCEs included smoking cessation, exercise, and health literacy on diet. In order to validate the OSCE scoring, the rating was done by two independent trained evaluators. The scenario and ratings were calibrated with a group of faculty modeling the encounter. Faculty reviewed tapes with residents and provided feedback.

### Self-assessments

Improvement in lifestyle medicine skills and behaviors was also measured with baseline and follow-up self-assessment surveys after the completion of their lifestyle medicine practicum rotation. The survey included topics such as the different approaches used to promote healthy lifestyle, rate of lifestyle counseling, barriers to lifestyle counseling, knowledge and confidence in lifestyle medicine, and personal health behaviors. The questions were answered on a scale of 1 to 10, with 1 being the least confident or knowledgeable to 10 being the most confident or knowledgeable.

### Statistical analysis

*T*-tests and paired *T*-tests were used to assess surveys taken at baseline and follow-up, and for assessment of OSCE scores. Statistical significance was set at an alpha of 0.05. Data were analyzed with the use of SAS software for Windows (version 9.3; SAS Institute, Cary, NC). Results were expressed as means and standard deviations in text and tables. This study was considered exempt by the Griffin Hospital Institutional Review Board.

## Results

A total of 20 preventive medicine residents participated in one or more components of the lifestyle medicine training at Griffin Hospital between 2010 and 2013. All residents completed a baseline survey and 10 completed the follow-up survey. Program participants included 55% women, 65% of Asian ethnicity, and 85% aged over 30 years (Table 2). The lifestyle medicine lecture series was very well attended by residents with over 95% rating the lectures good or excellent during the 2010–2013 academic years. Ten residents participated in the lifestyle medicine rotation at ILM and all have rated the rotation excellent. It consistently received the highest evaluation of any rotation completed by the residents. From 20 residents, two completed the rotation at the IMC and three completed the rotation at the Yale-Griffin Prevention Research Center.

**Fig. 1:**
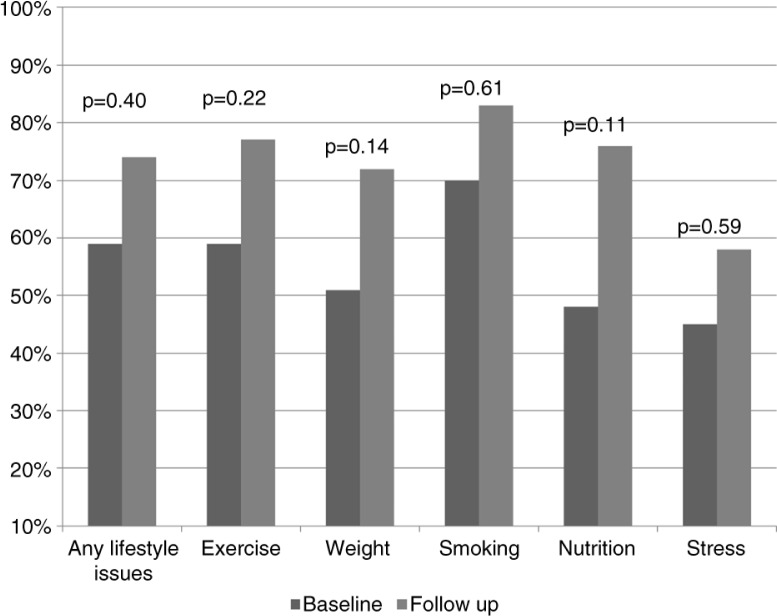
Lifestyle medicine outcomes. Residents’ self-reported topics discussed with their patient.

**Table 2:** Resident demographics, N=20

Gender	
Female	11 (55%)
Ethnicity	
Hispanic	1 (5%)
Race	
White	6 (30%)
Asian	13 (65%)
Other	1 (5%)
Age (years)	
20–30	3 (15%)
31–40	17 (85%)

**Table 3:** Baseline and follow-up survey results

		Baseline		Follow-up		
				
Question	Category	Mean	SD	Mean	SD	*p*
With what percentage of your patients do you currently discuss?	Any lifestyle issues	59%	0.24	74%	0.18	0.40
	Exercise	59%	0.23	77%	0.15	0.22
	Weight	51%	0.24	72%	0.22	0.14
	Smoking	70%	0.27	83%	0.14	0.61
	Nutrition	48%	0.22	76%	0.14	0.11
	Stress	45%	0.25	58%	0.31	0.59
How much do the following factors inhibit your lifestyle counseling of patients? 1=does not inhibit; 10=completely inhibits	Limited time	6.17	2.55	7.3	2.26	0.40
	Lack of reimbursement and other incentives	2.83	2.76	4.3	3.43	0.35
	Lack of knowledge/skills	3.42	1.83	4	1.63	0.78
	Lack of tools and materials	4.08	2.31	5.4	1.43	0.33
	Perceived poor patient compliance	4.58	1.78	6	2.26	0.12
Rate your current knowledge of each of the following: 1=not knowledgeable; 10=very knowledgeable	Exercise and physical activity	7.58	1.38	8.33	0.87	0.13
	Weight	7.83	1.03	8.33	0.87	0.20
	Smoking	8.17	1.34	8.56	0.88	0.35
	Nutrition	7.17	1.75	8.11	0.97	0.23
	Stress	6.75	1.71	7.78	0.97	0.24
How confident are you in discussing the following topics with your patients? 1=not confident; 10=very confident	Exercise and physical activity	8.17	1.8	8.6	1.08	0.14
	Weight	8.08	1.78	8.5	1.18	0.13
	Smoking	8.5	1.73	8.8	1.14	0.65
	Nutrition	6.89	2.09	8.2	1.14	0.47
	Stress	7.5	2.02	7.7	1.16	0.51
Rate the following items from 1 to 10 based on your personal health behaviors	Concerns of one's weight	5.58	2.19	6.4	2.59	0.44
	Current eating habits	6.92	2.31	7.2	1.81	0.34
	Managing your own stress	6.08	2.15	7	1.56	0.03

**Fig. 2:**
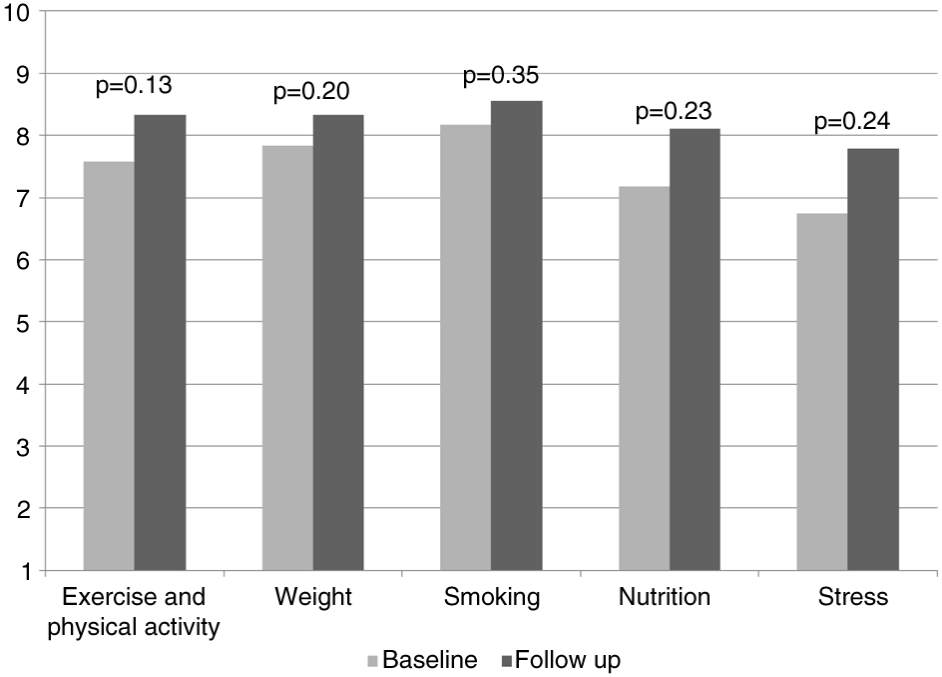
Residents’ knowledge. Residents’ self-reported knowledge on the following topics ranked from 1 (not knowledgeable at all) to 10 (very knowledgeable).

**Fig. 3:**
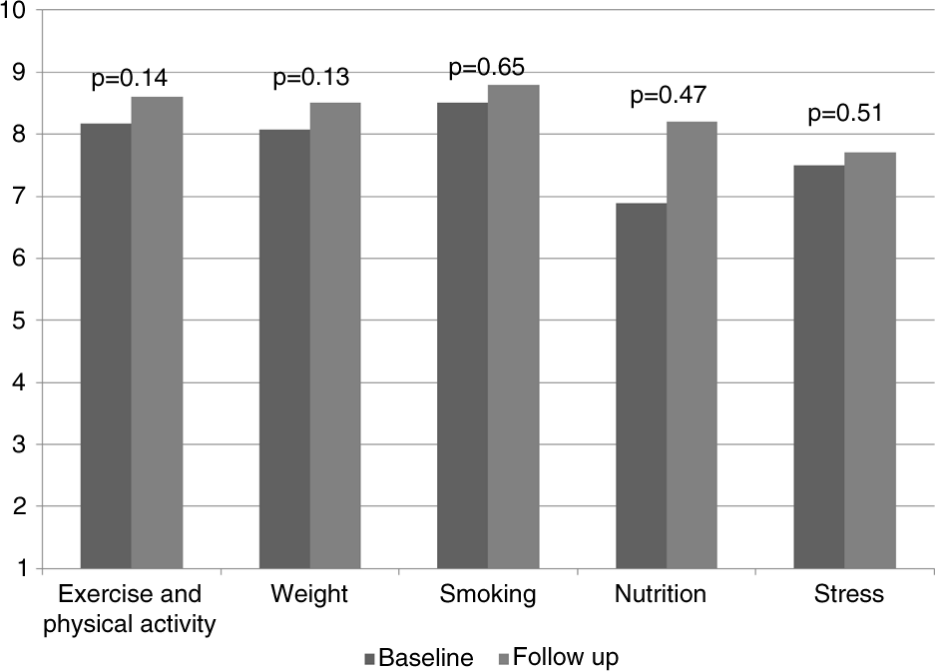
Residents’ confidence. Residents’ confidence in discussing the following topics with their patients ranked from 1 (no confidence) to 10 (very confident).

**Fig. 4:**
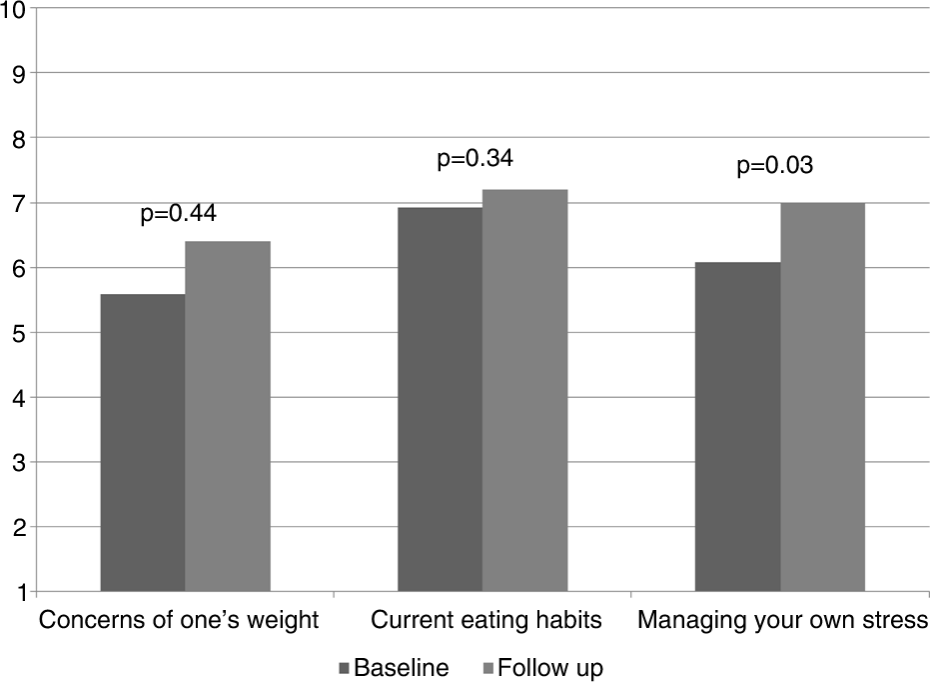
Residents’ self-reported health behaviors. Residents’ perception of weight is ranked from 1 (underweight) to 10 (overweight); eating habits is ranked from 1 (unhealthy eating habits) to 10 (very healthy eating habits); and managing stress is ranked from 1 (unmanageable) to 10 (very manageable).

There was about a 15% increase in residents' discussion of lifestyle issues with their patients based on their baseline and follow-up surveys, but results did not reach statistical significance due to small sample size (Table 3 and Fig. 1). There was a slight but statistically insignificant increase in the residents’ knowledge on various lifestyle components as well as their confidence in discussing lifestyle issues with patients (Table 3, Figs. 2 and 3). Finally, we did see a slight trend toward improvement in residents’ own health behaviors (Fig. 4). Residents’ self-reported quality of their eating habits went from a score of 6.9 prior to the rotation to 7.2 post-rotation in ILM (on a scale of 1 to 10, with a score of 1 depicting the worst quality and 10 depicting the highest quality), but results did not reach statistical significance. Concern for one's weight was 5.6 pre-rotation and 6.4 post-rotation, and finally the greatest and statistically significant change was seen in the residents’ management of stress with a score of 6.1 which increased to 7 post-rotation (Table 3 and Fig. 4).

**Fig. 5:**
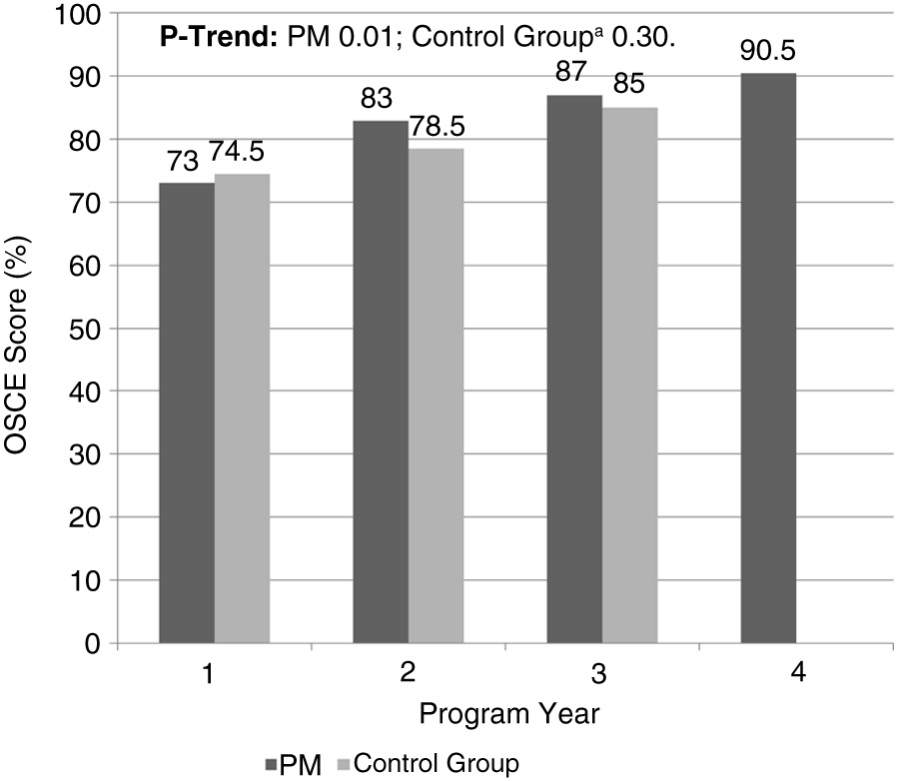
Change in OSCE by program year. ^a^Control group included residents in internal medicine residency program at the same institution.

Twenty residents completed the OSCEs prior to initiating this curriculum and scored an average of 60%. The performance of preventive medicine residents on OSCEs increased each year they were in the program (average OSCE score: PGY1 73%, PGY2 83%, PGY3 87%, and PGY4 91%, *p*=0.01 for trend) (see Table 3 and Fig. 5). Our internal medicine and preliminary residents served as a control, since they did participate in didactics but not in lifestyle medicine rotations. Internal medicine and preliminary residents who completed the same OSCEs had a slightly lower average score (76%) compared with preventive medicine residents (80%). However, this difference did not reach statistical significance (*p*=0.11).

## Discussion

Lifestyle medicine has gained increasing importance in the US healthcare system, and physicians and other health care providers are considered key sources of information and advice for patients on how to adopt and sustain a healthy lifestyle. However, there is a lack of organized curriculum in residency training to prepare future physicians for lifestyle medicine counseling. This paper provides initial results from a lifestyle medicine curriculum for our preventive medicine residency program at a community-based teaching hospital.

An interesting phenomenon we noted was that residents who were exposed to these curricula showed a trend toward improvement in their own health behaviors. Prior studies demonstrate that there is positive correlation between preventive health habits of physicians and their patients (21). Therefore, we consider this a significant development and a positive by-product of this curriculum, which will likely benefit patients in the long run.

The study should be interpreted in the light of its strengths and limitations. Due to the small size of our residency program, only a limited number of residents have completed this training. We faced several challenges in our quest to operationalize this curriculum. There were increased demands on faculty and residency staff to spend significant time in scheduling various components of this training. Since the rotation at the ILM was more than 2.5 hours away from Griffin Hospital, we had to arrange for accommodation for our residents at a significant cost. We were fortunate to have grant support which mostly absorbed the travel and boarding costs. We found that grouping two residents together decreased scheduling problems as well as the cost of sharing hotel rooms, and it increased the efficiency of the faculty at ILM. Residents also liked the idea of going together as they were able to bounce ideas of each other and were more interactive during rotations. Although we had planned to record actual resident–patient (with patient consent) encounters during ambulatory clinic visits for lifestyle counseling–related visits, this was not feasible due to legal concerns over videotaping. Despite limitations, our results showed a modest improvement and positive trend toward improvement in residents’ attitudes. Our results, based on 3 years of experience with this innovative curriculum, demonstrated a modest increase in the knowledge of lifestyle medicine components among our residents. Residents also demonstrated increased self-perceived efficacy in counseling patients. Prior studies showed that physicians often note lack of self-efficacy in behavioral health risk counseling as a barrier to counseling (5, 6). Other strengths of the curriculum are the broad mix of training methods and an objective evaluation of the program. Finally, the uniqueness of this training program stands out. To our knowledge, this is the first training program of its kind and there are no prior published studies on evaluation of the lifestyle medicine curriculum for residency programs.

The improvement in residents’ knowledge and self-reported comfort in discussing lifestyle medicine topics was at best modest and perhaps lower than expected and did not reach statistical significance due to small sample size. This could also be explained by the fact that there were only six didactics sessions per year and the ILM rotation offered was for only 2 weeks, due to constraints in our curriculum and other competing curricular demands. OSCEs showed improvement by program year in preventive medicine and preliminary medicine/internal medicine but were only statistically significant in the preventive medicine group. We hypothesize that increasing the amount of rotation time and number of didactics would demonstrate further improvement in knowledge and competency of residents.

We have demonstrated that a curriculum in lifestyle medicine is feasible. Residency programs with emphasis on preventive medicine and primary care have several resources available to design and replicate this curriculum. Lifestyle medicine competencies have been published to help guide design of the training program (1). Residents view the lifestyle medicine rotation positively as they can benefit from this rotation personally and apply lessons learned to their practice with immediate effect. Residents can learn key skills such as motivational interview skills and the use of exercise prescriptions, and we do hope that other residency programs will be able to find local resources for training their residents in lifestyle medicine. We believe that this curriculum is feasible and replicable.

## Conclusion

Despite the limitations, we feel that we have been able to demonstrate that a curriculum in lifestyle medicine is feasible to implement and is largely acceptable to trainee residents. This curriculum needs further replication in other programs. Many programs may be able to find local partners to create and implement this curriculum.
